# Supplementary material for: EXOC6 (Exocyst Complex Component 6) Is Associated with the Risk of Type 2 Diabetes and Pancreatic β-Cell Dysfunction
Source: Biology (Basel). 2022 Mar 1;11(3):388. doi: 10.3390/biology11030388 (PMC8945791; doi:10.3390/biology11030388)
Supplement: Supplementary file 1 [file biology-11-00388-s001.zip › biology-1583200-SI.pdf]

Supplemnatry Figures

Impact of Exoc6/6b silencing on the expression of β-cell function genes. Total protein was extracted from Exoc6/6b-silenced cells or siRNA negative control after 48 h of transfection and subjected for western blot analysis for Pro/insulin, PDX1, NEUROD1, PDX1, GLUT2, GCK, INSβ and VAMP2 relative to the endogenous control protein β-actin in Exoc6-silenced cells or Exoc6b-silenced cells. Data were obtained from three independent experiments.

Figure S1:

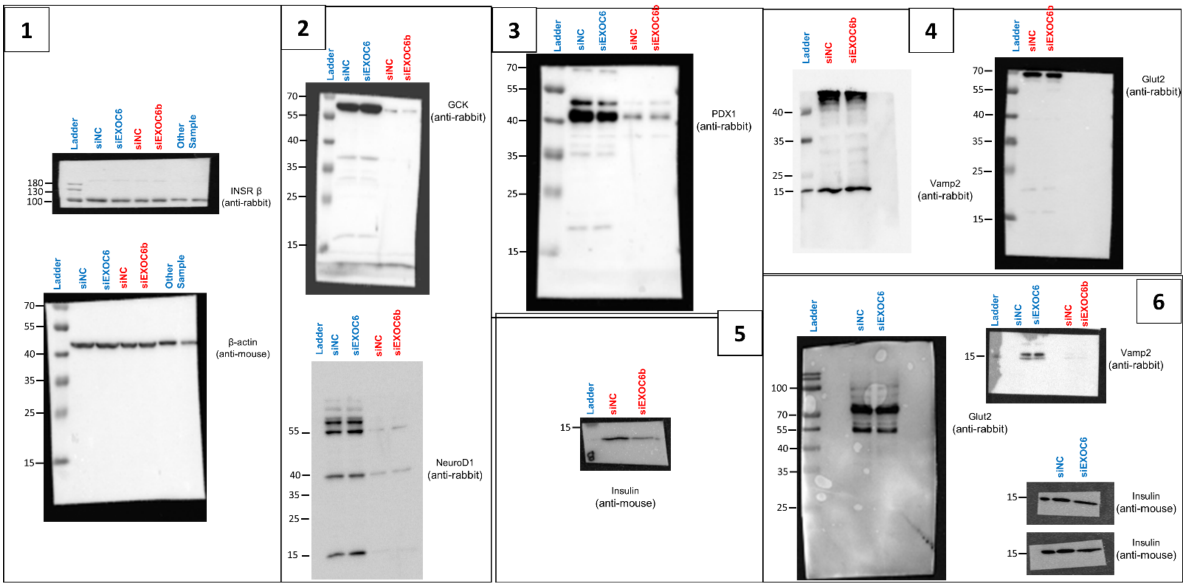

Figure S2:

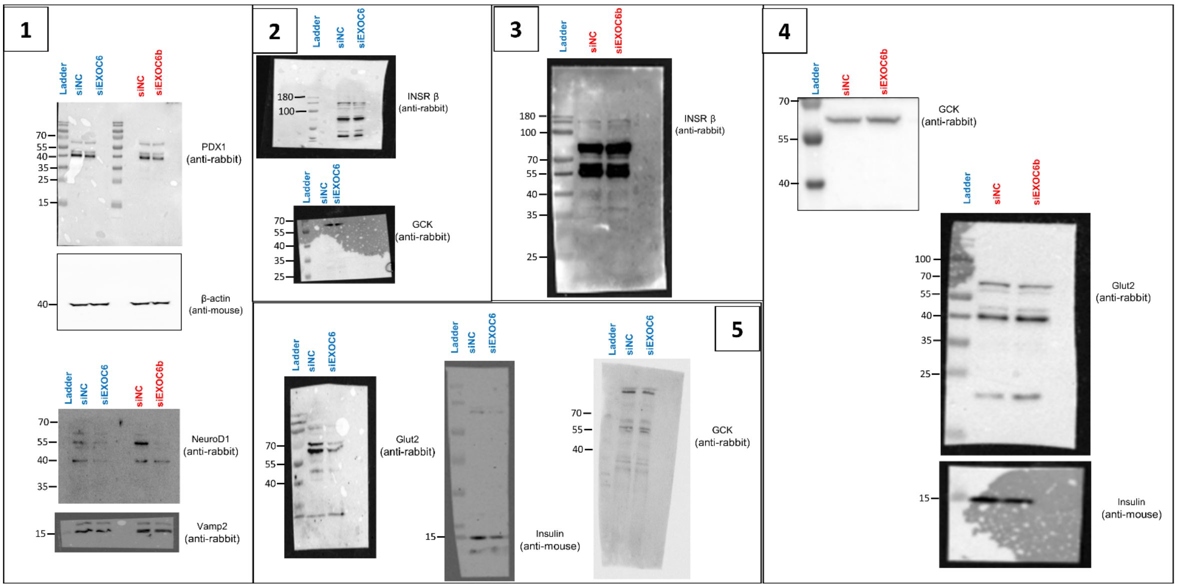

Figure S3:

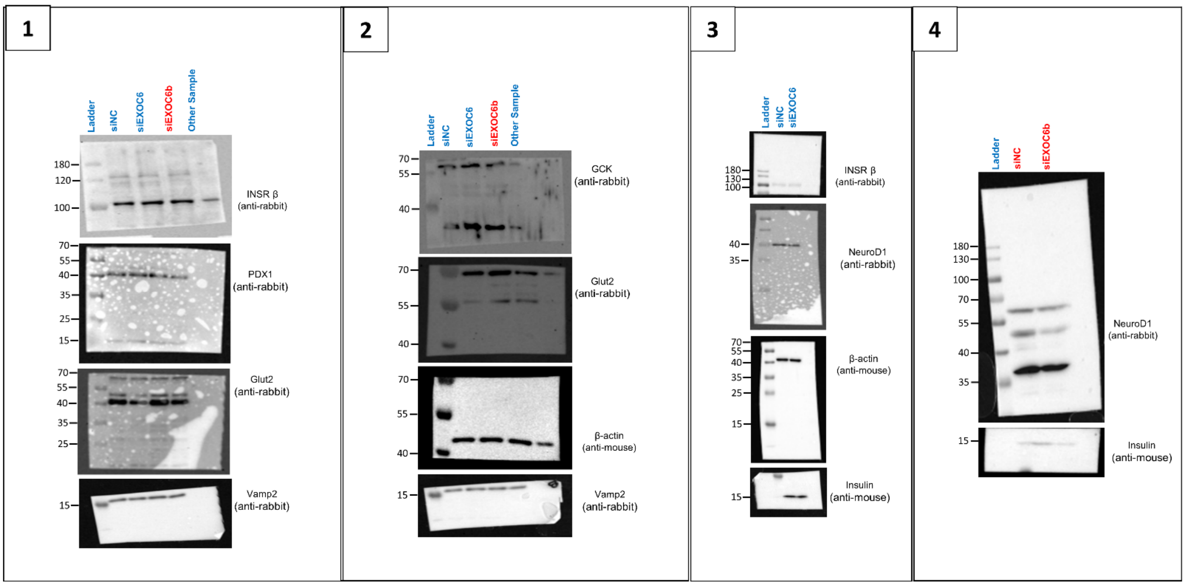
1
Ladder
siNC
siEXOC6
siEXOC6b
Other Sample
180
120
100
INSR β
(anti-rabbit)
70
55
40
35
25
15
PDX1
(anti-rabbit)
Glut2
(anti-rabbit)
Vamp2
(anti-rabbit)
2
GCK
(anti-rabbit)
Glut2
(anti-rabbit)
β-actin
(anti-mouse)
Vamp2
(anti-rabbit)
3
130
INSR β
(anti-rabbit)
NeuroD1
(anti-rabbit)
β-actin
(anti-mouse)
Insulin
(anti-mouse)
4
NeuroD1
(anti-rabbit)
Insulin
(anti-mouse)
